# Conceptual study on extraction of formic acid from the electrolyte after electroreduction of CO_2_: Desalination and dehydration using a high-silica chabazite zeolite membrane

**DOI:** 10.1016/j.heliyon.2023.e20259

**Published:** 2023-09-18

**Authors:** Norihiro Suzuki, Rumi Katsukawa, Naoya Ishida, Yuta Shiroma, Tsugumi Kagaya, Takeshi Kondo, Makoto Yuasa, Chiaki Terashima, Akira Fujishima

**Affiliations:** a Research Center for Space System Innovation, Research Institute for Science and Technology, Tokyo University of Science, 2641 Yamazaki, Noda, Chiba, 278-8510, Japan; b Carbon Value Research Center, Research Institute for Science and Technology, Tokyo University of Science, 2641 Yamazaki, Noda, Chiba, 278-8510, Japan; c Department of Pure and Applied Chemistry, Faculty of Science and Technology, Tokyo University of Science, 2641 Yamazaki, Noda, Chiba, 278-8510, Japan

**Keywords:** Zeolite membrane, Pervaporation, Desalination, Dehydration, Molecular sieve effect

## Abstract

Here, we propose a two-step pervaporation system with a high-silica CHA (chabazite) membrane, which has sufficient resistance to water and acid, to demonstrate the extraction and condensation of the formic acid formed by electroreduction of CO_2_. The kinetic diameters of water and formic acid are similar and smaller than the pore size of CHA, while the hydrated electrolyte ions (e.g., K^+^ and Cl^−^) are larger than the pore size of CHA. Consequently, the electrolyte ions are separated from the mixture of water and formic acid in the first desalination process, and then water molecules are easily removed from the mixture in the second dehydration process. From 300 ml of an approximately 3 wt% formic acid aqueous solution containing 0.5 M KCl, 10 ml of 18.2 wt% formic acid was obtained.

## 1 Introduction

To cope with rapid climate change, suppression of CO_2_ emissions is an urgent issue. Therefore, various strategies toward carbon neutrality, which is the concept of zero net CO_2_ emissions, have been proposed, and scientific and technological trials have been performed worldwide. To realize carbon neutrality while maintaining economic activities, using CO_2_ as a carbon source of chemicals is important. If the amount of CO_2_ consumed as a product source exceeds the amount of CO_2_ emitted during economic activities, a carbon negative society is even achievable.

Among the chemicals that can be produced from CO_2_, we focus on formic acid. Formic acid is used in silage, the tanning and textile industries, pharmaceuticals, and food chemicals [1]. Formic acid is also attractive as the liquid-hydrogen energy carrier owing to its high volumetric capacity (53 g H_2_/L) and low toxicity [2]. With a suitable iridium-based complex, formic acid decomposes to H_2_ (and CO_2_) at moderate temperatures (60–80 °C) and normal pressure [[3], [4], [5]].

To produce formic acid (and/or formate) using renewable energy, such as solar light, light-driven CO_2_ reduction with a heterogeneous photocatalyst [6], a Z-scheme heterostructure-coupled photosystem [7], a Fe-based metal–organic framework [8], a mixed metal (Zr/Ti), a mixed-ligand metal–organic framework [9], and an enzyme immobilized in a light-harvesting scaffold [10] have been reported. Electrochemical CO_2_ reduction is also a good candidate if electricity generated by renewable energy is used. Therefore, electrodes [[11], [12], [13]] and electrocatalysts [[14], [15], [16], [17], [18], [19], [20], [21]] with high Faraday efficiency, low over potential, high current density, and long-term stability for conversion of formic acid (formate) have been extensively investigated. However, because electroreduction of CO_2_ is performed in a liquid electrolyte, the formed formic acid is diluted with the electrolyte. Therefore, extraction and condensation of formic acid is required to realize social implementation. Although the use of a solid-state electrolyte has recently been proposed [22,23], to the best of our knowledge, effective separation of formic acid from a conventional liquid electrolyte and subsequent condensation have not been reported.

Distillation is a simple and well-known separation method. However, because the boiling point of formic acid (100.8 °C) is close to that of water (100 °C) at normal pressure, when the composition of formic acid reaches 77.6 wt% in the formic acid–water system, an azeotrope boils at 107.6 °C under normal pressure. Therefore, a formic acid aqueous solution denser than 77.6 wt% cannot be directly extracted by simple distillation [1], and other separation methods that do not depend on the gas–liquid phase equilibrium should be considered.

Solvent extraction is a useful and classical separation method. During the BASF process, which is an industrial formic-acid-production process, a secondary amide is used to extract formic acid (and some of the water). The extract is distilled in a dehydration column, and then sufficient water is distilled to obtain the required formic acid concentration in the pure-acid column, which is performed in vacuo [1]. However, this process requires large-scale facilities and has high thermal cost, and the ammine should be properly treated so that it does not affect the environment. Other separation methods, such as reverse osmosis, electrodialysis, and diffusion dialysis, cannot sufficiently separate formic acid and the electrolyte. Although ion exchange can remove the electrolyte, it cannot concentrate formic acid.

Considering the above-mentioned information, we chose a zeolite membrane to achieve simple and organic-solvent-free formic acid extraction. Separation with a zeolite membrane is based on the molecular sieve effect and selective adsorption effect, and gas separation [[24], [25], [26], [27], [28], [29], [30], [31], [32], [33], [34]], separation between isomers [35,36], dehydration of organic solvents [[37], [38], [39], [40], [41]], and desalination [[42], [43], [44], [45], [46], [47], [48]] have been investigated using zeolite membranes. NaA-type zeolite (Linde type-A zeolite with sodium ions) membranes are commercially used for dehydration because the pore size of Linde type-A zeolite (0.41 nm) [49] is larger than the kinetic diameter of water (0.296 nm) [49], and the membrane is hydrophilic owing to the low Si/Al ratio. However, NaA-type zeolite membranes are not durable under water-rich and acidic conditions. Therefore, in this study, we chose a high-silica chabazite (CHA) membrane [50], which has sufficient resistance to water and acid, and we investigated whether desalination and dehydration of formic acid formed in the electrolyte was possible. Followed by elemental studies, we designed a simple system to extract formic acid formed in the electrolyte and a demonstrative study was conducted.

## 2 Experimental

### 2.1 Materials

**Fig. 1:**
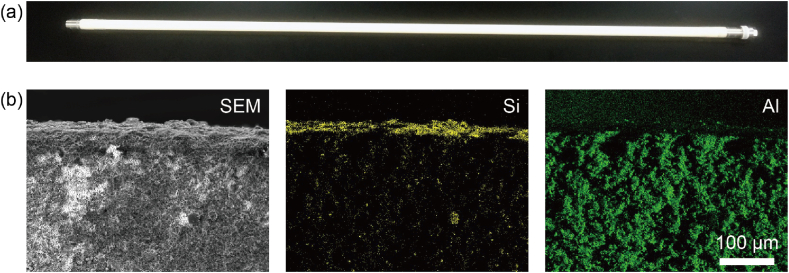
(a) Photograph of the zeolite membrane tube. (b) Cross-sectional scanning electron microscope (SEM) image and energy-dispersive X-ray mapping images of the membrane tube. Si is derived from the high-silica CHA membrane, while Al is mainly derived from the tubular porous alumina base.

A commercially available high-silica CHA zeolite membrane tube for industrial use (Si/Al = ∼10, ZEBREX ZX-1, Mitsubishi Chemical Corporation (Tokyo, Japan)) with a pore diameter of 0.38 nm [49] was chosen as the separation filter (Fig. 1(a)). A thin zeolite layer (approximately 25 μm thick) was formed on the tubular porous aluminum base (Fig. 1(b)).

Reagent grade (40 wt%) and industrial grade (76 wt%) formic acid were obtained from Wako Pure Chemical Corporation (Osaka, Japan) and Asahi Chemical (Osaka, Japan), respectively. Potassium chloride were purchased from FUJIFILM Wako Pure Chemical Corporation. All of the chemicals were used without further purification.

### 2.2 Pervaporation system

**Fig. 2:**
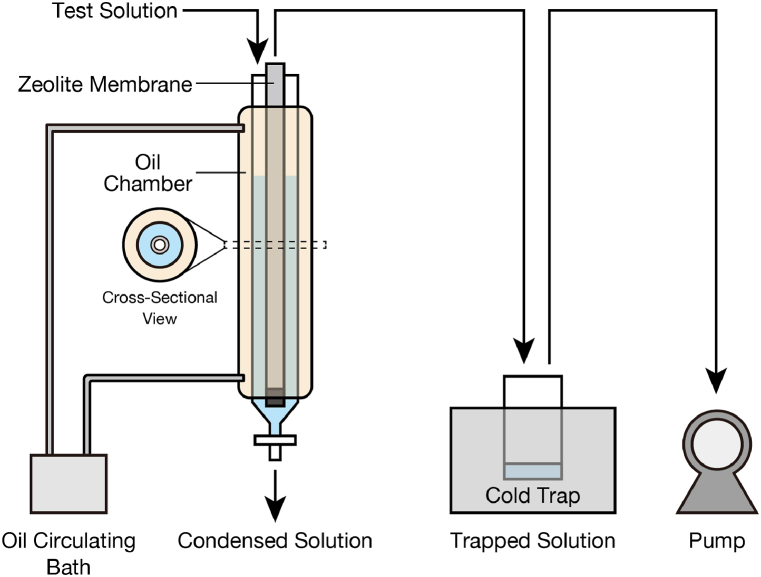
Schematic of the pervaporation system.

The pervaporation system used in the study is shown in Fig. 2. A zeolite membrane was set inside a glass zeolite membrane chamber, which was buried in a glass heating chamber, and heated silicone oil was circulated by a circulating bath during operation. The oil temperature was set to 90 °C. A faucet was attached to the bottom of the membrane chamber, through which the condensed solution was removed. The opening edge of the zeolite membrane was connected to a diaphragm pump to keep the inside the zeolite membrane (i.e., permeate side) at approximately 1 kPa. A glass condenser was set in the cooling trap to freeze the vapor that had passed through the zeolite membrane.

### 2.3 System operation and analysis

In the desalination test, 3 wt% formic acid aqueous solution containing 0.5 M KCl (i.e., the electrolyte for electrochemical CO_2_ reduction [11]) was used as the test solution. Although electrochemical CO_2_ reduction is frequently conducted in basic electrolytes such as KHCO_3_ and NaHCO_3_ aqueous solution, most of the formed formic acid existed as ions (HCOO^-^) in basic solutions. In contrast, because KCl aqueous solution is weakly acidic (probably due to the dissolved CO_2_), the electrolyte will become more acidic as formed formic acid increases, and then the formic acid tends to exist as molecules (HCOOH). The test solution was fed into the space between the oil chamber and the zeolite membrane in the membrane chamber. Formic acid aqueous solutions (without KCl) with various concentrations were used for the dehydration tests.

Qualitative and quantitative analysis of the potassium and chlorine ions in the test, condensed, and trapped solutions was performed with an ion chromatograph system (Dionex ICS-1100, Thermo Fisher Scientific). Before the measurements, the test and trapped solutions were diluted 2500 times with deionized water, while the condensed solutions were diluted 7500 times with deionized water, and 1 ml of each solution was taken for the measurement. Dionex IonPac CS12A-8 μm (Thermo Fisher Scientific) and Dionex IonPac AS12A (Thermo Fisher Scientific) columns were used for the cations and anions, respectively. An aqueous solution of methanesulfonic acid (18 mmol/L) and a mixture of Na_2_CO_3_ (2.7 mmol/L) and NaHCO_3_ (0.3 mmol/L) were used as the eluents for the cations and anions, respectively. The column temperature was set to 30 °C for both the cations and anions. The flow rates were 1.0 and 1.5 ml/min for the cations and anions, respectively. A Dionex ERS500 electrolytically regenerated suppressor was used to decrease the electric conductivity of the eluent and improve the sensitivity for the target ions. The suppressor current was set to 30 and 60 mA for the cations and anions, respectively.

Neutralization titration with sodium hydroxide, in which 1.0 w/v% phenolphthalein ethanol(90) solution was used as an indicator, was performed to calculate the concentration of the formic acid aqueous solution (*X*, wt%) by eq. (1):

(1) $$\frac{M_{HCOOH}∙C_{NaOH}∙\frac{V_{NaOH}}{1000}}{W}\times 100=X$$

where *M*_*HCOOH*_, *C*_*NaOH*_, *V*_*NaOH*_, and *W* are the molar mass of formic acid (46.025 g/mol), concentration of the sodium hydroxide aqueous solution (in this study, 0.50 mol/L), volume of the sodium hydroxide aqueous solution used in neutralization titration (mL), and weight of the test solution (g), respectively.

The volume of formic acid *V*_*HCOOH*_ (ml) was calculated by eq. (2):

(2) $$\frac{d_{HCOOH}∙V_{HCOOH}}{d_{HCOOH}∙V_{HCOOH}+d_{H_{2}O}∙\left(V-V_{HCOOH}\right)}\times 100=X$$

where *d*_*HCOOH*_, *d*_*H2O*_, and *V* are the density of formic acid (1.22 g/ml), density of water (1.00 g/ml), and volume of the formic acid aqueous solution (mL), respectively.

The collection rate (%), which indicates the effectiveness without adsorption loss of formic acid in the zeolite membrane, chamber, and other components, was calculated by eq. (3):

(3) $$Collection\;Rate\;\left(\%\right)=\frac{\left(Volume\;of\;Formic\;Acid\;in\;Condenced\;Solution\right)+\left(Volume\;of\;Formic\;Acid\;in\;Trapped\;Solution\right)}{Volume\;of\;Formic\;Acid\;in\;Test\;Solution}\times 100$$

The passing rate (%), which indicates how much formic acid flows through the membrane, was calculated by eq. (4):

(4) $$Passing\;Rate\;\left(\%\right)=\frac{Volume\;of\;Formic\;Acid\;in\;Trapped\;Solution}{\left(Volume\;of\;Formic\;Acid\;in\;Condenced\;Solution\right)+\left(Volume\;of\;Formic\;Acid\;in\;Trapped\;Solution\right)}\times 100$$

## 3 Results and discussion

### 3.1 Desalination of an electrolyte containing formic acid

**Fig. 3:**
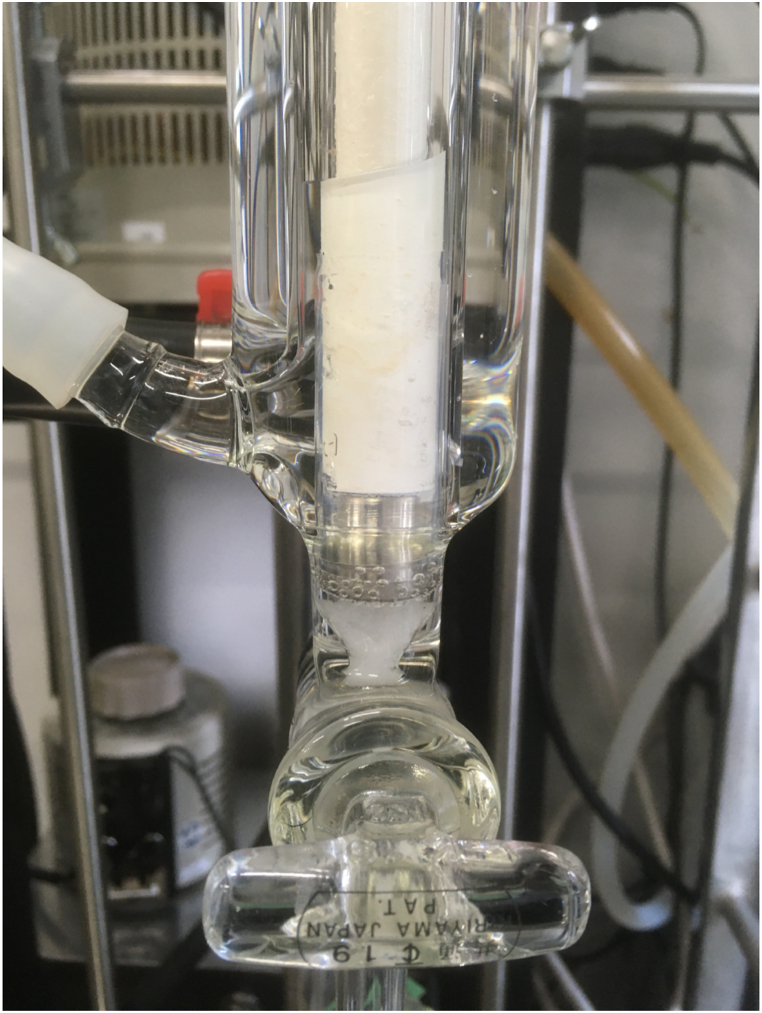
Photograph of the white precipitate at the bottom of the zeolite membrane chamber.

During electrochemical reduction of CO_2_, the formed formic acid accumulates in the electrolyte. Therefore, a desalination process is required before the dehydration process to obtain condensed formic acid. Previous studies have reported that desalination by pervaporation can be achieved with NaA- [42] and Mobil-5-type zeolite membranes [[43], [44], [45], [46], [47]], which have larger pore sizes than CHA zeolite. Therefore, desalination with a CHA zeolite membrane should easily occur. Desalination of the test solution (3 wt% formic acid aqueous solution containing 0.5 M KCl) using the CHA zeolite membrane was investigated. As the operation proceeded, a precipitate was observed at the bottom of the zeolite membrane chamber (Fig. 3), suggesting that the ions could not penetrate the zeolite membrane and were deposited as a salt on the bottom of the zeolite membrane chamber. A high salt concentration enough to make such precipitate damages the zeolite membrane. Therefore, a moderate operation not to precipitate the salt is important during the desalination process and the membrane withstood after around 300 h operation.

**Fig. 4:**
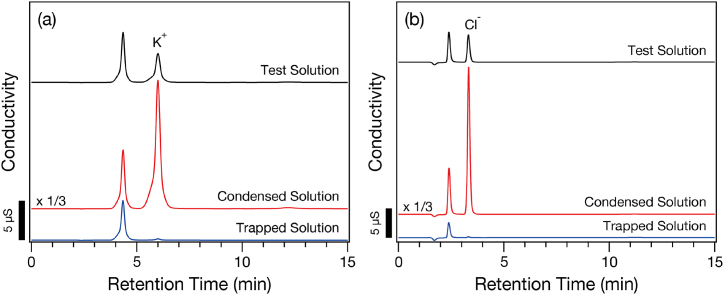
(a) Cation and (b) anion chromatograms of the test, condensed, and trapped solutions. The prominent peaks that appear at early retention time in (b) are derived from formic acid.

The test, condensed, and trapped solutions were analyzed by ion chromatography. The cation and anion chromatographs are shown in Fig. 4 (a) and Fig. 4 (b), respectively. K^+^ and Cl^−^ ions were not detected in the trapped solution. Because ions are hydrated in aqueous solution and the hydrated diameters of K^+^ and Cl^−^ (0.662 nm for K^+^ and 0.664 nm for Cl^−^) [51] are much larger than the pore size of CHA zeolite (0.38 nm), these ions cannot penetrate the zeolite membrane.

As shown in eq. (5), formic acid and formate are in equilibrium in aqueous solution:

(5) $$\text{HCOOH}+H_{2}O\;⇄\;{\text{HCOO}}^{-}+H_{3}O^{+}\;K=\frac{\left[{HCOO}^{-}\right]\left[{H_{3}O}^{+}\right]}{\left[HCOOH\right]\left[H_{2}O\right]}=const\text{.}$$

Assuming a large excess water, because [H_2_O] is almost constant,

(6) $$K_{a}=K\left[H_{2}O\right]=\frac{\left[{HCOO}^{-}\right]\left[{H_{3}O}^{+}\right]}{\left[HCOOH\right]}=const.∴{pK}_{a}=-\log\;K_{a}=const\text{.}$$

where *K*_a_ is the acid dissociation constant, and p*K*_*a*_ = 3.75 for formic acid. The pH (the potential of hydrogen) is expressed as

(7) $$pH=-\log\left[{H_{3}O}^{+}\right]$$

Thus, from eqs. (6), (7), the ratio between formic acid and formate is expressed as

(8) $$-\log\;K_{a}=-\log\left(\frac{\left[{HCOO}^{-}\right]\left[{H_{3}O}^{+}\right]}{\left[HCOOH\right]}\right)∴\frac{\left[{HCOO}^{-}\right]}{\left[HCOOH\right]}=10^{\left(pH-pK_{a}\right)}=10^{\left(pH-3.75\right)}$$

Because the pH of the test solution was 1.8, judging from eq. (8), most of the formic acid existed as molecules. The kinetic diameter of the formic acid molecule is approximately 0.4 nm [52], which is similar to the pore size of CHA zeolite, and thus formic acid molecules can penetrate the zeolite membrane. Indeed, a considerable amount of formic acid was detected in the trapped solution (Table 1). The collection rate was calculated to be 94%, showing that the loss of formic acid during operation of the system was negligible. Calculation of the passing rate showed that 72% of the formic acid molecules passed through the membrane and transferred to the trapped solution. Therefore, pervaporation with a CHA zeolite membrane is useful for desalination of the electrolyte after electrochemical CO_2_ reduction.

**Table 1:** Amounts of formic acid and electrolyte ions (K^+^ and Cl^−^) in each solution after the pervaporation process. Owing to the volume of the glass condenser set in the cooling trap, the trapped solution was separately taken.

	Feed solution volume (ml)	Collected solution volume (ml)	Concentration of formic acid (wt%)	Amount of formic acid (ml)	Amount of K^+^ (g)	Amount of Cl^−^(g)
Test Solution	490	–	3.1	12.4	8.81	7.85
Condensed Solution	–	29	13.6	3.3	10.35	10.20
Trapped Solution 1	–	240	1.6	3.1	0.01	0.09
Trapped Solution 2	–	198	3.3	5.3	0.06	0.02

### 3.2 Dehydration of dilute formic acid aqueous solution

Next, the ability of the CHA zeolite membrane for dehydration of formic acid was investigated. Because the kinetic diameter of water is much smaller than that of formic acid and the pore size of CHA zeolite, water molecules are expected to predominantly pass through the zeolite membrane, resulting in condensation of formic acid in the zeolite membrane chamber. The results of dehydration of an approximately 3 wt% formic acid aqueous solution by pervaporation are summarized in Table 2. The concentration of the obtained condensed formic acid was approximately 40 wt%, which is the concentration of industrial grade formic acid. However, the calculated passing rates indicated that only 30%–50% of the formic acid remained in the chamber. Therefore, to collect the formic acid that passed through the membrane, the trapped solution was fed into the chamber, and the dehydration process was repeatedly performed. By collecting the condensed solutions, 23.3 ml of solution containing 3.1 ml of formic acid was obtained (Table 3), which was 79% of the formic acid in the initial test solution. In other words, from 150 ml of an approximately 3 wt% formic acid aqueous solution, 23.3 ml of 16.0 wt% formic acid aqueous solution was obtained after 9 h.

**Table 2:** Volume of formic acid (FA) and its concentration in each solution after the pervaporation process. The collection and passing rates are also given.

	Test Solution			Condensed Solution			Trapped Solution			Collection Rate (%)	Passing Rate (%)	Operation Time (h)
	Volume of solution (ml)	Concentration of FA (wt%)	Volume of FA (ml)	Volume of solution (ml)	Concentration of FA (wt%)	Volume of FA (ml)	Volume of solution (ml)	Concentration of FA (wt%)	Volume of FA (ml)			
#1	130	3.1	3.3	3.9	37.0	1.3	91	1.9	1.4	81	53	4.0
#2	199	3.2	5.2	3.8	44.0	1.5	157	2.5	3.2	89	68	5.0
#3	265	3.2	6.9	5.0	42.8	1.9	214	2.3	4.1	86	68	4.5

**Table 3:** Volume of formic acid (FA) and its concentration in each solution after the initial operation and re-condensation process using the trapped solution. The collection and passing rates are also given.

	Test Solution			Condensed Solution			Trapped Solution			Collection Rate (%)	Passing Rate (%)	Operation Time (h)
	Volume of solution (ml)	Concentration of FA (wt%)	Volume of FA (ml)	Volume of solution (ml)	Concentration of FA (wt%)	Volume of FA (ml)	Volume of solution (ml)	Concentration of FA (wt%)	Volume of FA (ml)			
Initial condensation (dehydration) operation												
	150	3.2	3.9	4.8	27.8	1.1	145	2.2	2.6	96	69	2.0
Re-condensation of formic acid with trapped solution												
1st	144	2.2	2.6	4.2	20.5	0.7	139	1.6	1.9	101	72	2.0
2nd	138	1.6	1.8	5.4	14.3	0.6	131	1.1	1.2	100	65	1.75
3rd	130	1.1	1.2	3.8	11.3	0.4	123	0.8	0.8	97	69	1.75
4th	122	0.8	0.8	5.1	6.4	0.3	116	0.6	0.6	106	67	1.5

To investigate whether further condensation was possible, the denser formic acid solution was used as the test solution. The results are given in Table 4. Formic acid denser than 77.6 wt%, which is the azeotrope of water/formic acid at atomic pressure, was obtained, indicating the excellent performance of the system for distillation of formic acid. During the repetitive dehydration of 40 wt% formic acid, there was no damage on the CHA membranes in 72.5 h operation.

**Table 4:** Volume of formic acid (FA) and its concentration in each solution after the pervaporation process starting from the dense FA solution. The collection and passing rates are also given.

	Test Solution			Condensed Solution			Trapped Solution			Collection Rate (%)	Passing Rate (%)	Operation Time (h)
	Volume of solution (ml)	Concentration of FA (wt%)	Volume of FA (ml)	Volume of solution (ml)	Concentration of FA (wt%)	Volume of FA (ml)	Volume of solution (ml)	Concentration of FA (wt%)	Volume of FA (ml)			
#1	64	39.5	22.3	18	78.9	13.6	46	20.7	8.1	97	37	3.0
#2	64	78.3	47.8	30	91.0	26.8	32	60.4	17.8	93	40	3.0

### 3.3 Sequential operation for pure formic acid extraction

**Fig. 5:**
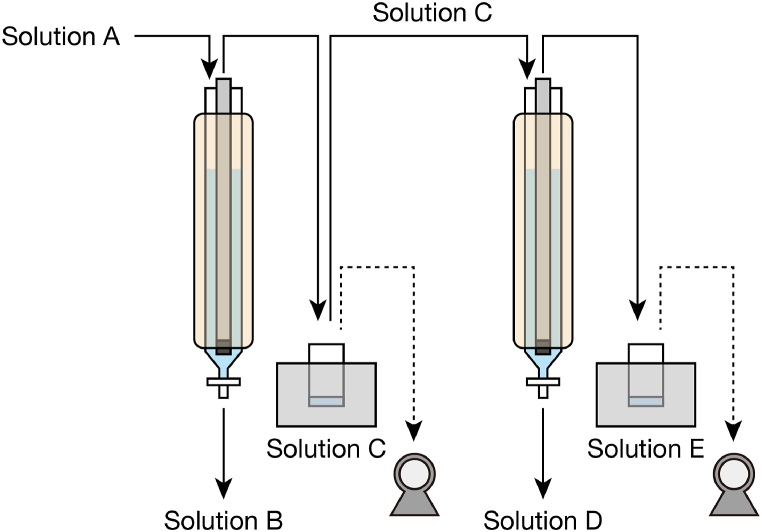
Schematic of sequential operation for extraction of pure formic acid.

Because both desalination and dehydration are possible by pervaporation with a CHA zeolite membrane, isolation and purification of formic acid through sequential operation was investigated. A schematic of the system is shown in Fig. 5. In the first step (i.e., desalination), an approximately 3 wt% formic acid aqueous solution containing 0.5 M KCl was used as the test solution (solution A), and the condensed solution (solution B) and trapped solution (solution C) were collected. In the second step (i.e., dehydration), solution C was used as the test solution, and the condensed solution (solution D) and trapped solution (solution E) were collected. The estimated amounts of the ions are given in Table 5. Neither the cation nor the anion were detected in solutions C, D, and E, confirming that desalination was perfectly performed. Through dehydration of solution C, 10 ml of 18.2 wt% formic acid aqueous solution (solution D) was obtained (Table 6). Therefore, a series of pervaporation steps with a CHA zeolite membrane is a simple way to extract and condense the formic acid formed during electroreduction of CO_2_.

**Table 5:** Amounts of electrolyte ions (K^+^ and Cl^−^) in each solution during sequential operation estimated by ion chromatography.

	Amount of K^+^ (g)	Amount of Cl^−^ (g)
Solution A	5.78	4.97
Solution B	5.01	4.15
Solution C	Not Detected	Not Detected
Solution D	Not Detected	Not Detected
Solution E	Not Detected	Not Detected

**Table 6:** Volume of formic acid (FA) and its concentration in each solution during sequential operation. The collection and passing rates are also given.

	Test Solution			Condensed Solution			Trapped Solution			Collection Rate (%)	Passing Rate (%)	Operation Time (h)
	Volume of solution (ml)	Concentration of FA (wt%)	Volume of FA (ml)	Volume of solution (ml)	Concentration of FA (wt%)	Volume of FA (ml)	Volume of solution (ml)	Concentration of FA (wt%)	Volume of FA (ml)			
1st Step	(Solution A)			(Solution B)			(Solution C)			91	63	3.0
	300	3.2	7.9	25	12.6	2.6	272	2.0	4.5			
2nd Step	(Solution C)			(Solution D)			(Solution E)			100	65	3.0
	267	2.0	4.5	10	18.2	1.5	256	1.4	2.9			

## 4 Conclusions

Using the molecular sieve effect of a zeolite membrane, we have demonstrated a method to isolate and purify the formic acid formed by electrochemical CO_2_ reduction in an electrolyte. Because the pore size of CHA zeolite and kinetic diameter of formic acid are similar, pervaporation with the zeolite membrane can achieve both desalination (in which flowing of the formic acid to the cold trap is required) and dehydration (in which maintaining the formic acid in the membrane chamber for condensation is required).

Because this work is primitive, there are several points should be overcome to realize scaled-up system for practical application. One is a material of zeolite membrane chambers. In this study, a glass chamber was used due to its high chemical tolerance. However, because a glass is fragile, mechanically robust metal chamber is much suitable for the large-scale system and glass lining (i.e., surface modification of metal container with glass coating) is a promising way to introducing chemical stability on metal chambers.

Another point is a low efficiency in dehydration process. As shown in Table 2, during the dehydration of 3 wt% of formic acid, large part of formic acid passed through the zeolite membrane and only 30%–50% of formic acid remained in the chamber. To prevent an outflow of formic acid, decreasing an affinity of formic acid for membrane surface by changing more hydrophilic membrane would be effective.

Although there is much room for improvement in this study, it will be a gateway to practical application of electroreduction of CO_2_ to formic acid.

## Author contributions

Norihiro Suzuki: Conceived and designed the experiments; Analyzed and interpreted the data; Wrote the paper.

Rumi Katsukawa: Performed the experiments; Analyzed and interpreted the data.

Naoya Ishida: Analyzed and interpreted the data.

Yuta Shiroma, Tsugumi Kagaya: Performed the experiments.

Takeshi Kondo, Makoto Yuasa: Contributed regents, materials, analysis tools or data.

Chiaki Terashima, Akira Fujishima: Conceived and designed the experiments; Analyzed and interpreted the data; Contributed regents, materials, analysis tools or data

## Additional information

No additional information is available for this paper.

## Declaration of competing interest

The authors declare that they have no known competing financial interests or personal relationships that could have appeared to influence the work reported in this paper.

## References

[1] Hietala J., Vuori A., Johnsson P., Pollari I., Reutemann W., Kieczka H. (2016).

[2] Eppinger J., Huang K.-W. (2017). Formic acid as a hydrogen carrier. ACS Energy Lett..

[3] Onishi N., Ertem M.Z., Xu S., Tsurusaki A., Manaka Y., Muckerman J.T., Fujita E., Himeda Y. (2016). Direction to practical production of hydrogen by formic acid dehydrogenation with Cp*Ir complexes bearing imidazoline ligands. Catal. Sci. Technol..

[4] Iguchi M., Himeda Y., Manaka Y., Kawanami H. (2016). Development of an iridium-based catalyst for high-pressure evolution of hydrogen from formic acid. ChemSusChem.

[5] Onishi N., Kanega R., Fujita E., Himeda Y. (2019). Carbon dioxide hydrogenation and formic acid dehydrogenation catalyzed by iridium complexes baring pyridyl-pyrazole ligands: effect of an electron-donating substituent on the pyrazole ring on the catalytic activity and durability. Adv. Synth. Catal..

[6] Kuriki R., Sekizawa K., Ishitani O., Maeda K. (2015). Visible-light-driven CO_2_ reduction with carbon nitride: enhancing the activity of ruthenium catalysts. Angew. Chem. Int. Ed..

[7] Wang Y., Shang X., Shen J., Zhang Z., Wang D., Lin J., Wu J.C.S., Fu X., Wang X., Li C. (2020). Direct and indirect Z-scheme heterostructure-coupled photosystem enabling cooperation of CO_2_ reduction and H_2_O Oxidation. Nat. Commun..

[8] Wang D., Huang R., Liu W., Sun D., Li Z. (2014). Fe-based MOFs for photocatalytic CO_2_ reduction: role of coordination unsaturated sites and dual excitation pathways. ACS Catal..

[9] Lee Y., Kim S., Kang J.K., Cohen S.M. (2015). Photocatalytic CO2 reduction by a mixed metal (Zr/Ti), mixed ligand metal-organic framework under visible light irradiation. Chem. Commun..

[10] Chen Y., Li P., Zhou J., Buru C.T., Ɖorđević L., Li P., Zhang X., Cetin M.M., Stoddart J.F., Stupp S.I., Wasielewski M.R., Farha O.M. (2020). Integration of enzymes and photosensitizers in a hierarchical mesoporous metal-organic framework for light-driven CO_2_ reduction. J. Am. Chem. Soc..

[11] Natusi K., Iwakawa H., Ikemiya N., Nakata K., Einaga Y. (2018). Stable and highly efficient electrochemical production of formic acid from carbon dioxide using diamond electrodes. Angew. Chem. Int. Ed..

[12] Wang J., Wang H., Han Z., Han J. (2015). Electrodeposited porous Pb electrode with improved electrocatalytic performance for the electroreduction of CO_2_ to formic acid. Front. Chem. Sci. Eng..

[13] Dai C., Sun L., Song J., Liao H., Fisher A.C., Xu Z.J. (2019). Selective electroreduction of carbon dioxide to formic acid on cobalt-decorated copper thin films. Small Methods.

[14] Bai X., Chen W., Zhao C., Li S., Song Y., Ge R., Wei W., Sun Y. (2017). Exclusive Formation of formic acid from CO_2_ electroreduction by a tunable Pd-Sn alloy. Angew. Chem. Int. Ed..

[15] Zhang J., Yin R., Shao Q., Zhu T., Huang X. (2019). Oxygen vacancies in amorphous InO_x_ nanoribbons enhance CO_2_ adsorption and activation for CO_2_ electroreduction. Angew. Chem. Int. Ed..

[16] Lu P., Tan X., Zhao H., Xiang Q., Liu K., Zhao X., Yin X., Li X., Hai X., Xi S., Wee A.T.S., Pennycook S.J., Yu X., Yuan M., Wu J., Zhang G., Smith S.C., Yin Z. (2021). Atomically dispersed indium sites for selective CO2 electroreduction to formic acid. ACS Nano.

[17] Kortlever R., Peters I., Koper S., Koper M.T.M. (2015). Electrochemical CO_2_ reduction to formic acid at low overpotential and with high faradaic efficiency on carbon-supported bimetallic Pd-Pt nanoparticles. ACS Catal..

[18] Liu S., Lu X.F., Xiao J., Wang X., Lou X.W.D. (2019). Bi_2_O_3_ nanosheets grown on multi-channel carbon matrix to catalyze efficient CO_2_ electroreduction to HCOOH. Angew. Chem. Int. Ed..

[19] Klinkova A., Luna P.D., Dinh C.-T., Voznyy O., Larin E.M., Kumacheva E., Sargent E.H. (2016). Rational design of efficient palladium catalysts for electroreduction of carbon dioxide to formate. ACS Catal..

[20] Duan Y.-X., Zhou Y.-T., Yu Z., Liu D.-X., Wen Z., Yan J.-M., Jiang Q. (2021). Boosting production of HCOOH from CO2 electroreduction via Bi/CeO_x_. Angew. Chem. Int. Ed..

[21] Geng W., Chen W., Li G., Dong X., Song Y., Wei W., Sun Y. (2020). Induced CO_2_ electroreduction to formic acid on metal-organic frameworks via node deping. ChemSusChem.

[22] Xia C., Zhu P., Jiang Q., Pan Y., Liang W., Stavitski E., Alshareef H.N., Wang H. (2019). Continuous production of pure liquid fuel solutions via electrocatalytic CO_2_ reduction using solid-electrolyte devices. Nat. Energy.

[23] Fan L., Xia C., Zhu P., Lu Y., Wang H. (2020). Electrochemical CO2 reduction to high-concentration pure formic acid solutions in an all-solid-state reactor. Nat. Commun..

[24] Kusakabe K., Kuroda T., Murata A., Morooka S. (1997). Formation of a Y-type zeolite membrane on a porous α-alumina tube for gas separation. Ind. Eng. Chem. Res..

[25] Aoki K., Kusakabe K., Morooka S. (2020). Separation of gases with an A-type zeolite membrane. Ind. Eng. Chem. Res..

[26] Nikolakis V., Xomeritakis G., Abibi A., Dickson M., Tsapatsis M., Vlachos D.G. (2001). Growth of a faujasite-type zeolite membrane and its application in the separation of saturated/unsaturated hydrocarbon mixtures. J. Membr. Sci..

[27] Tomita T., Nakayama K., Sakai H. (2004). Gas separation characteristics of DDR type zeolite membrane. Microporous Mesoporous Mater..

[28] Bernal M.P., Coronas J., Menéndez M., Santamaría J. (2004). Separation of CO_2_/N_2_ mixtures using MFI-type zeolite membranes. AIChE J..

[29] Giannakopoulos I.G., Nikolakis V. (2005). Separation of propylene/propane mixtures using faujasite-type zeolite membranes. Ind. Eng. Chem. Res..

[30] Cui Y., Kita H., Okamoto K. (2004). Preparation and gas separation performance of zeolite T membrane. J. Mater. Chem..

[31] Gu X., Dong J., Nenoff T.M. (2005). Synthesis of defect-free FAU-type zeolite membranes and separation for dry and moist CO_2_/N_2_ mixtures. Ind. Eng. Chem. Res..

[32] Himeno S., Tomita T., Suzuki K., Nakayama K., Yajima K., Yoshida S. (2007). Synthesis and permeation properties of a DDR-type zeolite membrane for separation of CO_2_/CH_4_ gaseous mixtures. Ind. Eng. Chem. Res..

[33] Krishna R., van Baten J.M. (2010). *In Silico* screening of zeolite membranes for CO_2_ capture. J. Membr. Sci..

[34] Korelskiy D., Ye P., Fouladvand S., Karimi S., Sjöberg E., Hedlund J. (2015). Efficient ceramic zeolite membranes for CO_2_/H_2_ separation. J. Mater. Chem. A.

[35] Lai Z., Bonilla G., Diaz I., Nery J.G., Sujaoti K., Amat M.A., Kokkoli E., Terasaki O., Thompson R.W., Tsapatsis M., Vlachos D.G. (2003). Microstructural optimization of a zeolite membrane for organic vapor separation. Science.

[36] Choi J., Jeong H.-K., Snyder M.A., Stoeger J.A., Masel R.I., Tsapatsis M. (2009). Grain boundary defect elimination in a zeolite menbrane by rapid thermal processing. Science.

[37] Gallego-Lizon T., Edwards E., Lobiundo G., dos Santos L.F. (2002). Dehydration of water/*t*-butanol mixtures by pervaporation: comparative study of commercially available polymeric, microporous silica and zeolite membranes. J. Membr. Sci..

[38] Urtiaga A., Gorri E.D., Casado C., Ortiz I. (2003). Pervaporative dehydration of industrial solvents using a zeolite NaA commercial membrane. Sep. Purif. Technol..

[39] Sawamura K., Furuhata T., Sekine Y., Kikuchi E., Subramanian B., Matsukata M. (2015). Zeolite membrane for dehydration of isopropylalcohol-water mixture by vapor permeation. ACS Appl. Mater. Interfaces.

[40] Chen X., Wang J., Yin D., Yang J., Lu J., Zhang Y., Chen Z. (2013). High-performance zeolite T membrane for dehydration of organics by a New varying temperature hot-dip coating method. AIChE J..

[41] Liu D., Zhang Y., Jiang J., Wang X., Zhang C., Gu X. (2015). High-performance NaA zeolite membranes supported on four-channel ceramic hollow fibers for ethanol dehydration. RSC Adv..

[42] Wang L., Yang J., Wang J., Raza W., Liu G., Lu J., Zhang Y. (2020). Microwave synthesis of NaA zeolite membranes on coarse microporous α-Al_2_O_3_ tubes for desalination. Microporous Mesoporous Mater..

[43] Li L., Dong J., Nenoff T.M., Lee R. (2004). Desalination by reverse osmosis using MFI zeolite membranes. J. Membr. Sci..

[44] Kazemimoghadam M., Mohammadi T. (2007). Synthesis of MFI zeolite membranes for water desalination. Desalination.

[45] Zhu B., Kim J.H., Na Y.-H., Moon I.-S., Connor G., Maeda S., Morris G., Gray S., Duke M. (2013). Temperature and pressue effects of desalination using a MFI-type zeolite membrane. Membranes.

[46] Zhu B., Hong Z., Milne N., Doherty C.M., Zhou L., Lin Y.S., Hill A.J., Gu X., Duke M. (2014). Desalination of seawater ion complexes by MFI-type zeolite membranes: temperature and long term stability. J. Member. Sci..

[47] Zhu B., Myat D.T., Shin J.-W., Na Y.-H., Moon I.-S., Connor G., Maeda S., Morris G., Gray S., Duke M. (2015). Application of robust MFI-type zeolite membrane for desalination of saline wastewater. J. Member. Sci..

[48] Cao Z., Zeng S., Xu Z., Arvantis A., Yang S., Gu X., Dong J. (2018). Ultrathin ZSM-5 zeolite nanosheet laminated membrane for high-flux desalination of concentrated brines. Sci. Adv..

[49] Bowen T.C., Noble R.D., Falconer J.L. (2004). Fundamentals and applications of pervaporation through zeolite membranes. J. Membr. Sci..

[50] Sato K., Sugimoto K., Shimotsuma N., Kikuchi T., Kyotani T., Kurata T. (2012). Development of practically available up-scaled high-silica CHA-type zeolite membranes for industrial purpose in dehydration of *N*-methyl pyrrolidone solution. J. Membr. Sci..

[51] Nightingale E.R. (1959). Phenomenological theory of ion solvation. Effective radii of hydrated ions. J. Phys. Chem..

[52] Jae J., Tompsett G.A., Foster A.J., Hammond K.D., Auerbach S.M., Lobo R.F., Huber G.W. (2011). Investigation into the shape selectivity of zeolite catalysts for biomas conversion. J. Catal..

